# Assessment of the real MDT teaching model in medical imaging internship: an educational-oriented study

**DOI:** 10.1186/s13244-026-02364-8

**Published:** 2026-08-14

**Authors:** Qingling Yang, Jian Xu, Tianji Wang, Rui An, Minwen Zheng, Yayu Huang, Fan Guo, Jing Ren

**Affiliations:** 1 Department of Interventional Surgery Center, Xijing Hospital, The Fourth Military Medical University, Xi’an, China; 2 Department of Radiology, Xijing Hospital, The Fourth Military Medical University, Xi’an, China; 3 Internal Medicine Teaching and Research Office, Xijing Hospital, The Fourth Military Medical University, Xi’an, China

**Keywords:** Multidisciplinary team (MDT), Radiology, Education (medical undergraduate), Clinical reasoning

## Abstract

**Objectives:**

To investigate whether the real multidisciplinary team (MDT) teaching mode can improve the learning effectiveness of interns majoring in medical imaging.

**Materials and methods:**

Prospective data from 58 medical imaging interns from July 2023 to January 2024 in a tertiary care radiology department were included. All interns were randomly assigned to either the experimental group or the control group using a random number table, with 29 interns in each group. The control group received the traditional teaching mode, whereas the experimental group was taught using a real MDT teaching model. Following the internship, the theoretical knowledge, practical reading skills, and clinical reasoning and decision test scores of the two groups were compared. Additionally, the teaching effectiveness and satisfaction of the two teaching modes were evaluated via questionnaires.

**Results:**

The practical skills (24.03 ± 1.18), clinical reasoning and decision (25.72 ± 1.85), and total score (84.41 ± 2.77) of the experimental group were significantly higher than those of the control group (23.21 ± 1.37), (24.17 ± 1.14), and (82.72 ± 2.45), respectively. However, there was no statistically significant difference in theoretical knowledge score between the two groups (34.66 ± 2.44 vs 35.34 ± 1.45, *p* = 0.197). The questionnaire survey results indicated that the experimental group had a more favorable evaluation of the real MDT teaching mode compared to the control group (*p* < 0.05). Moreover, the satisfaction rate of the experimental group with the teaching mode was significantly higher than that of the control group (χ^2^ = 12.301, *p* = 0.002).

**Conclusions:**

The real MDT teaching model improves interns’ clinical competencies, self-learning, teamwork, and overall educational satisfaction, showing promising utility in medical imaging training.

**Key Points:**

- **Question** The traditional teaching mode of radiology internship lacks interdisciplinary integration, which limits the clinical reasoning ability of interns.
- **Findings** Compared with the traditional teaching mode, the real MDT teaching mode in this study significantly improved the practical skills, clinical reasoning ability, and satisfaction of interns.
- **Critical relevance statement** The new interdisciplinary teaching model significantly improves the teaching effectiveness of radiology interns and helps cultivate their reasoning ability to combine imaging findings with clinical practice. This model is expected to be embedded in the intern training system.

**Graphical Abstract:**

**Figure:**
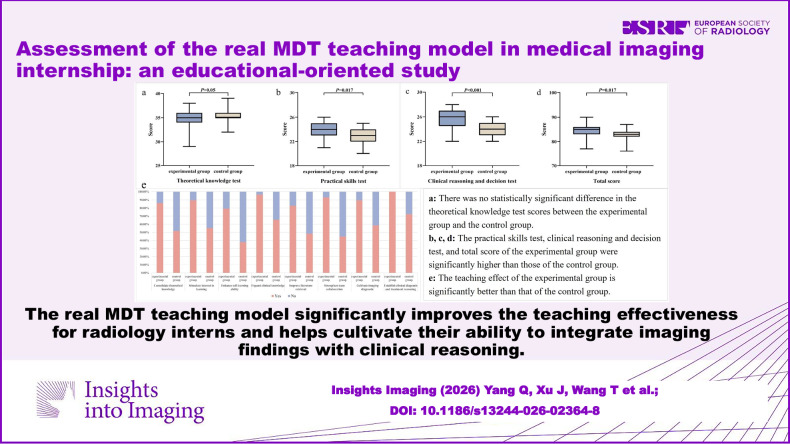

## Introduction

Medical imaging plays a pivotal role in modern healthcare, serving as the cornerstone for accurate diagnosis and treatment planning [1]. However, the rapid evolution of imaging technologies and the increasing complexity of multidisciplinary patient care necessitate that medical imaging education not only impart technical expertise but also cultivate clinical reasoning, interdisciplinary collaboration, and decision-making skills [2, 3]. Traditional radiology teaching models often emphasize passive knowledge acquisition through didactic lectures and isolated case reviews, which may not effectively bridge the gap between theoretical knowledge and real-world clinical practice [4, 5]. Within China’s medical education system, the final-year internship phase of a five-year undergraduate program serves as a critical bridge for medical students to integrate their theoretical knowledge with clinical practice, profoundly influencing their subsequent professional development [6].

Multidisciplinary team (MDT) approaches have been extensively implemented in clinical settings to optimize patient outcomes [7–10]. Radiologists play a crucial role in MDTs, providing their expertise in diagnostic imaging and interpretation [11–13]. By simulating real-world collaborative environments, MDT methods have shown promise in transforming medical education [14–16]. However, the integration of MDT-based teaching into radiology training remains underexplored, particularly in terms of skill acquisition and learner satisfaction. While existing studies have highlighted the benefits of MDT discussions in enhancing trainees’ diagnostic accuracy and clinical judgment [17], few have systematically assessed their impact on the development of comprehensive competencies, including practical skills, critical thinking, and teamwork.

This study aims to address this gap by comparing the effectiveness of a real MDT teaching model with traditional methods in medical imaging internships. We hypothesize that active participation in MDT discussions will significantly improve trainees’ practical skills, clinical decision-making abilities, and overall satisfaction, while maintaining foundational theoretical knowledge. By embedding interns in authentic clinical workflows, this study seeks to redefine radiology education paradigms and provide evidence-based strategies for nurturing adaptable, collaborative imaging specialists.

## Materials and methods

### Participants

This study selected 58 medical imaging interns who interned in the Department of Radiology at Xijing Hospital, Air Force Medical University, from July 2023 to January 2024 as the research subjects. All interns were randomly assigned to either the experimental group or the control group using a random number table, with 29 interns in each group. Written informed consent was obtained from all participants prior to the study, and approval was obtained from the Ethics Committee of Xijing Hospital, Air Force Medical University, with approval number KY202303056-1.

### Study design

**Fig. 1:**
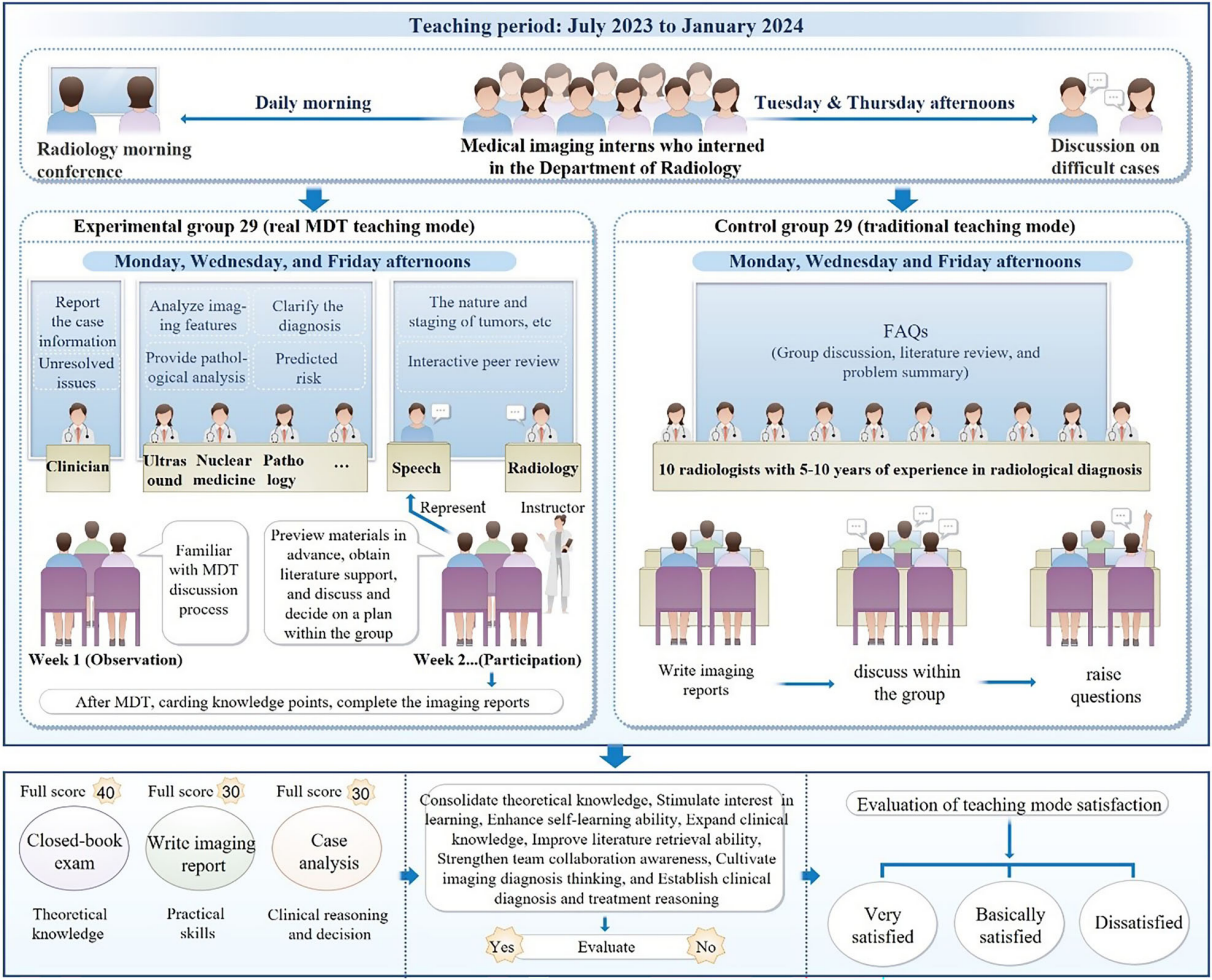
Detailed teaching process for two groups of interns

In this study, before commencing their internships, all interns were required to undergo a baseline assessment evaluating their theoretical knowledge, practical skills, and clinical reasoning and decision-making abilities. The experimental group engaged in the real MDT teaching model within the hospital, while the control group adhered to the traditional teaching model. Following the 6-month internship, both groups of interns underwent comprehensive professional knowledge assessments, which included tests on theoretical knowledge, practical skills, and clinical reasoning and decision-making. Additionally, interns were required to subjectively assess the effectiveness of the two teaching modes across eight dimensions and provide an overall satisfaction rate for each teaching method. See Fig. 1 for details.

### Teaching process

A detailed internship plan and content were developed in accordance with the requirements of the Medical Imaging Internship Outline, with a duration of 6 months. Both groups of students received a unified theoretical lecture of 2 class hours every morning, each lasting 40 min, amounting to a total of 140 class hours. In addition to these lectures, interns from both groups participated in departmental teaching activities, including the daily morning interpretation of imaging images and the discussion of difficult cases on Tuesday and Thursday afternoons.

The experimental group participated in real MDT teaching mode and engaged in MDT discussions within the hospital every Monday, Wednesday, and Friday afternoons. The MDT team consisted of senior-title physicians from radiology, ultrasound, nuclear medicine, pathology, and various clinical departments. The MDT discussion process was structured as follows:

1. Patient information reporting: the head physician from the relevant clinical department presented the patient’s basic information, including chief complaint, symptoms, current medical history, past medical history, diagnosis and treatment process, and the urgent problems to be addressed.
2. Imaging and diagnostic analysis: based on the medical history and imaging data, radiology experts analyzed the imaging features, proposed diagnostic results and differential diagnosis, and provided preliminary diagnosis and treatment recommendations. Experts in ultrasound and nuclear medicine followed a similar process. Pathology experts offered pathological analysis regarding classification and prognosis.
3. Comprehensive multidisciplinary analysis: clinical experts from various disciplines analyzed the cutting-edge research of the disease from their respective professional perspectives. They integrated the interpretations from imaging and pathology experts to clarify the diagnosis and formulate the next treatment plan, while also discussing potential risks during the treatment process. Throughout the discussion, experts from each discipline participated fully, and the supervising physician documented the entire process.

During the initial week, interns were scheduled to observe three MDT discussions to acclimate themselves to the process. In the subsequent week, they formally engaged in the discussions under the supervision of their mentors. It was imperative for interns to review the patient’s medical history, laboratory tests, imaging, and pathological data one day before the MDT meeting. They were required to conduct a literature-based analysis of the imaging findings and discuss within their group to formulate preliminary diagnostic and treatment recommendations. During each MDT discussion, an intern representative delivered a PowerPoint presentation, focusing on critical imaging information that guides clinical decision-making, such as the nature, staging, and involvement of surrounding tissues by the tumor, and proposed subsequent diagnostic and treatment steps. These presentations were then supplemented and refined by radiology experts. Following the MDT discussion, each intern reviewed the diagnostic and treatment concepts of the case using a mind map and independently completed the imaging report. Taking prostate cancer MDT as a case study, see Fig. S1 for details.

The control group was subjected to the traditional teaching method. On Monday, Wednesday, and Friday afternoons, 10 teachers, each with 5–10 years of experience in radiological diagnosis, led the interns in film reading and imaging report writing. During these sessions, the interns were permitted to engage in group discussions, consult relevant literature, and pose questions, which the teachers addressed promptly.

### Teaching evaluation

At the conclusion of the internship, a standardized assessment was administered in accordance with the requirements of the medical imaging internship syllabus. The evaluation encompassed three domains: theoretical knowledge, practical skills, and clinical reasoning and decision-making. Items with a calibrated difficulty of 0.7 were selected from an existing item pool to form a new assessment. This assembly yielded a test with an overall projected difficulty of 0.7 and a maximum score of 100. The theoretical knowledge examination was conducted in a closed-book format, comprising 10 single-choice questions and 1 short-answer question. This section aimed to evaluate the students’ retention and comprehension of fundamental medical imaging concepts. The maximum score for this section was 40 points, and participants were allotted 30 min to complete it. The practical skills assessment simulated the routine workflow of the radiology department. Participants were randomly assigned three cases from an imaging case repository, tasked with generating an imaging report, and subsequently submitting it for evaluation. The reports were scored by senior radiologists based on readability, completeness, and accuracy, thereby assessing the students’ diagnostic reasoning and film-reading proficiency. This section had a maximum score of 30 points, with a time limit of 30 min. The clinical reasoning and decision assessment required students to randomly select one case from a question bank. Based on the provided information, students were expected to verbally recount the medical history, symptoms, imaging findings, and laboratory results, formulate an imaging diagnosis and differential diagnoses, and propose subsequent diagnostic and treatment recommendations. The primary focus of this section was on clinical reasoning, with a maximum score of 30 points and a time limit of 10 min. This assessment was graded by two physicians holding associate senior or higher professional titles, and the average score was calculated.

Additionally, a survey questionnaire was distributed to all participants to evaluate the effectiveness of the two teaching modes across eight dimensions: consolidating theoretical knowledge, stimulating learning interest, enhancing self-learning ability, expanding clinical knowledge, improving literature retrieval ability, strengthening teamwork ability, cultivating imaging diagnosis thinking, and establishing clinical diagnosis and treatment reasoning. The evaluation outcomes were categorized into two options: ‘yes’ and ‘no’. The details of the questionnaire content are shown in Fig. S2.

Finally, interns from both groups were required to rate their satisfaction with their respective teaching modes. The criteria were divided into three levels: “very satisfied”, “basically satisfied”, and “dissatisfied”.

### Statistical analysis

All data were analyzed using SPSS version 26.0. Data distributions were evaluated with the Kolmogorov–Smirnov test. Normally distributed measurement data were presented as mean ± standard deviation $\left(\bar{x}\pm {{{\rm{s}}}}\right)$, and comparisons between groups were conducted using two independent sample t-tests. For measurement data that did not follow a normal distribution, medians [interquartile ranges (IQRs)] were used, and group comparisons were made with the Mann–Whitney *U*-test. Count data were expressed as frequency and percentage [cases (%)], and intergroup comparisons were carried out using chi-square (χ²) tests. A *p*-value of less than 0.05 was considered to indicate statistical significance.

## Results

### General information

The experimental group consisted of 29 interns, including 12 males and 17 females, with an average age of (22.24 ± 1.50) years. The control group consisted of 15 males and 14 females, with an average age of (22.69 ± 1.26) years. Both groups of interns had an undergraduate education level. There were no statistically significant differences (*p* > 0.05) in gender, age, and baseline assessment between the two groups, indicating comparability (Table 1).

**Table 1:** Participant general information in the two groups

Characteristics	Experimental group	Control group	*t*/ χ^2^	*p*-value
Number of participants	29	29		
Age $\left(\bar{x}\pm {{{\rm{s}}}}\right)$	22.24 ± 1.50 years (range 19–25 years)	22.69 ± 1.26 years (range 20–25 years)	−1.232	0.223
Gender [*n* (%)]			0.624	0.430
Male	12 (41.4%)	15 (51.7%)		
Female	17 (58.6%)	14 (48.3%)		
Baseline assessment score				
Theoretical knowledge	26.17 ± 1.65	26.55 ± 2.01	−0.786	0.435
Practical skills	18.00 ± 1.79	18.66 ± 1.45	−1.532	0.131
Clinical reasoning and decision	18.10 ± 1.90	18.21 ± 1.08	−0.255	0.800

### Professional knowledge test

**Fig. 2:**
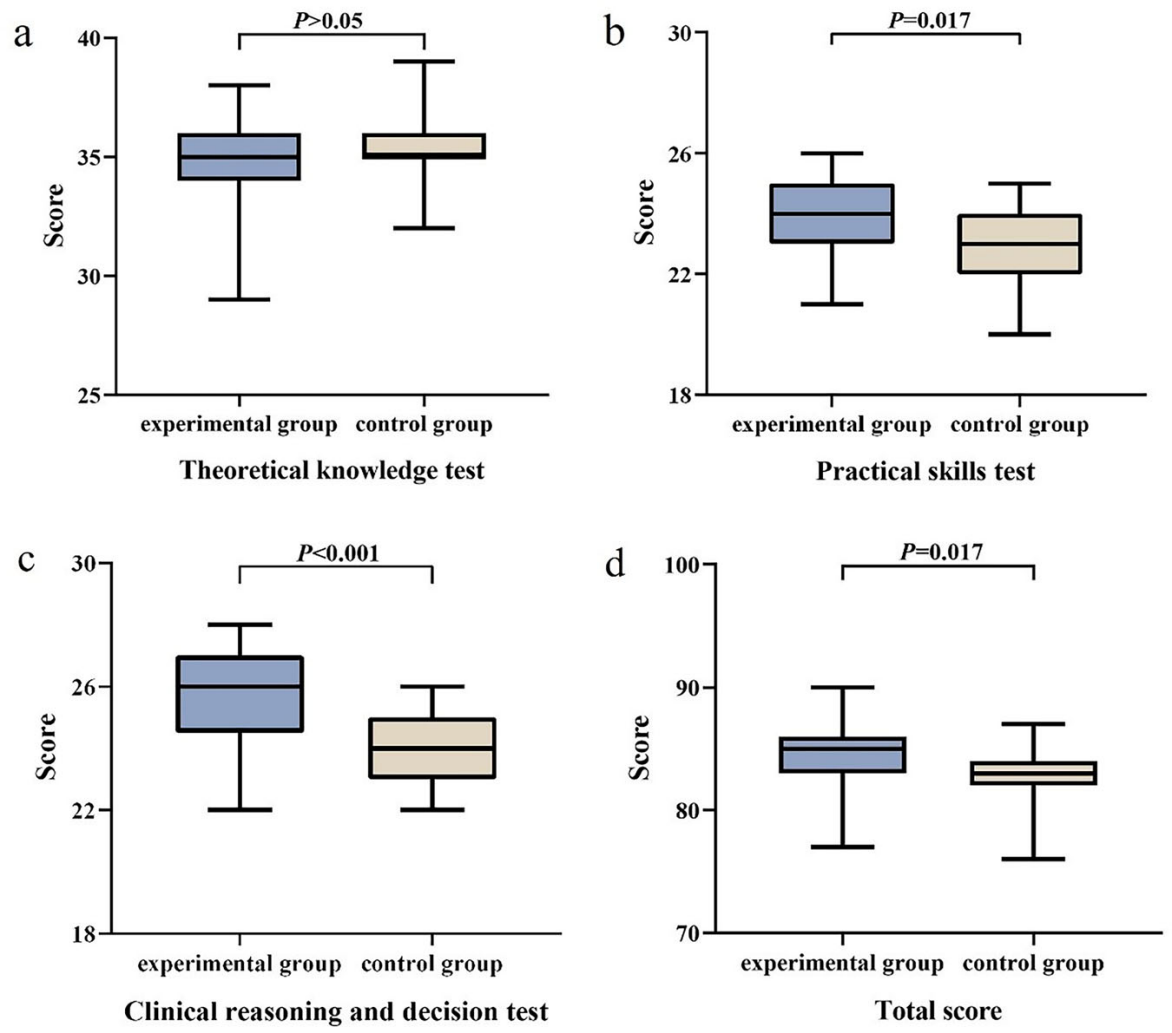
The box-plot showed that the experimental group had higher scores in the practical skills test (**b**), clinical thinking and decision test (**c**), and total score (**d**) than the control group, while there was no statistically significant difference in theoretical knowledge test scores (**a**) between the two groups

As shown in Table 2, compared with the control group, the experimental group showed higher scores in practical skills, clinical reasoning and decision, and total score (*p* < 0.05). The difference in theoretical knowledge test scores between the two groups was not statistically significant, as shown in Fig. 2.

**Table 2:** Comparison of test scores of the two groups of interns $\left(\bar{x}\pm {{{\rm{s}}}}\right)$

	Experimental group (*n* = 29)	Control group (*n* = 29)	*t*	*p*-value
Theoretical knowledge test (95% CI)	34.66 ± 2.44 (33.73–35.58)	35.34 ± 1.45 (34.79–35.89)	−1.310	0.197
Practical skills test (95% CI)	24.03 ± 1.18 (23.59–24.48)	23.21 ± 1.37 (22.68–23.73)	2.462	0.017
Clinical reasoning and decision test (95% CI)	25.72 ± 1.85 (25.02–26.43)	24.17 ± 1.14 (23.74–24.60)	3.850	< 0.001
Total score (95% CI)	84.41 ± 2.77 (83.36–85.47)	82.72 ± 2.45 (81.79–83.66)	2.461	0.017

*CI* confidence interval

### Teaching effectiveness evaluation

**Fig. 3:**
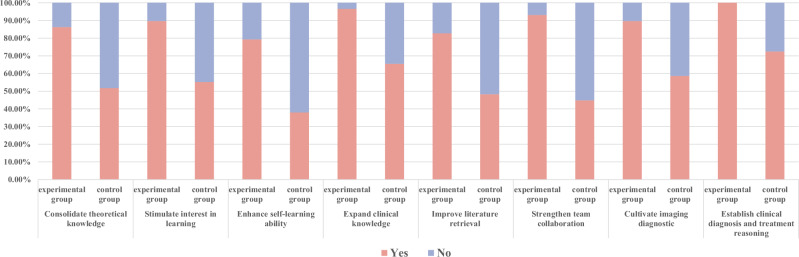
Comparison of the percentage of responses from two groups of participants to eight teaching effectiveness

The experimental group participants gave more positive evaluations of the eight items than the control group (Fig. 3). Compared with the traditional teaching mode of the control group, most people believed that the MDT teaching mode can achieve the corresponding teaching effectiveness more effectively, and the difference was statistically significant (Table 3).

**Table 3:** Comparison of teaching effectiveness evaluation between two groups [*n* (%)]

Evaluation item	Experimental group (*n* = 29)	Control group (*n* = 29)	*χ*^*2*^	*p-*value
Consolidate theoretical knowledge			6.340	0.012
Yes	24 (82.76)	15 (51.72)		
No	5 (17.24)	14 (48.28)		
Stimulate interest in learning			8.631	0.003
Yes	26 (89.66)	16 (55.17)		
No	3 (10.34)	13 (44.83)		
Enhance self-learning ability			10.235	0.001
Yes	23 (79.31)	11 (37.93)		
No	6 (20.69)	18 (62.07)		
Expand clinical knowledge			9.087	0.003
Yes	28 (96.56)	19 (65.52)		
No	1 (3.45)	10 (34.48)		
Improve literature retrieval ability			7.632	0.006
Yes	24 (82.76)	14 (48.28)		
No	5 (17.24)	15 (51.72)		
Strengthen team collaboration awareness			15.789	< 0.001
Yes	27 (93.10)	13 (44.83)		
No	2 (6.90)	16 (55.17)		
Cultivate imaging diagnostic thinking			7.284	0.007
Yes	26 (89.66)	17 (58.62)		
No	3 (10.34)	12 (41.38)		
Establish clinical diagnosis and treatment reasoning			9.280	0.002
Yes	29 (100.00)	21 (72.41)		
No	0 (0)	8 (27.59)		

### Teaching model satisfaction

The satisfaction rate of the experimental group participants with the teaching model was higher than that of the control group, and the difference was statistically significant (χ^2^ = 12.30, *p* = 0.002, Table 4).

**Table 4:** Comparison of satisfaction with teaching models between two groups of interns [*n* (%)]

Group	Very satisfied	Basically satisfied	Dissatisfied
Experimental group	20 (68.97%)	6 (20.69%)	3 (10.34%)
Control group	7 (24.14%)	11 (37.93%)	11 (37.93%)
*χ²*	12.301		
*p*-value	0.002		

## Discussion

In recent years, the MDT model has significantly optimized clinical diagnosis and treatment pathways by integrating the expertise of specialists from multiple fields. During this process, medical imaging serves as the “objective evidence chain” for disease diagnosis, providing a critical morphological and functional basis for MDT [11]. To offer diagnostic advice that truly aids patient management, radiology interns must understand the clinical context and integrate imaging information within the framework of clinical thinking [18]. However, traditional imaging education often confines students to “using images to analyze images”, and the provided imaging information frequently lacks correlation with clinical symptoms, laboratory tests, and treatment needs, thereby limiting its clinical guidance value [19]. Fortunately, the MDT teaching model based on rehearsal has been proven to be effective in cultivating students’ thinking abilities. By assuming different roles within the MDT, students can broaden their understanding of various diseases [16, 17, 20]. Therefore, our study introduces the real MDT teaching model into the radiology internship training system. Through multidisciplinary discussions of real cases, cross-validation of clinical, imaging, and pathological findings, and participation in the entire diagnostic and treatment process, we aim to enhance interns’ ability to integrate diverse information, break through disciplinary barriers, and establish an imaging diagnostic thinking oriented toward clinical need.

In this study, the real MDT teaching model was found to be more effective than traditional teaching models, yielding positive feedback. Interns in the experimental group achieved higher scores in practical skills, clinical reasoning, and decision assessment compared to those in the control group. The real MDT teaching mode emphasizes interdisciplinary collaboration, allowing interns to absorb relevant knowledge from various disciplines during cross-disciplinary discussions, understand the needs of clinical physicians, and analyze each image from a clinical perspective [14, 16]. In addition, structured reflection through mind maps and iterative feedback from multidisciplinary experts may enhance interns’ metacognitive skills and foster their imaging diagnostic thinking abilities [21]. Implementing the MDT diagnosis and treatment mode during medical education provides medical students with a robust platform for learning general medicine and lays a solid foundation for improving the efficiency of clinical disease diagnosis and treatment. Research has demonstrated that the MDT model can enhance evaluation outcomes and overall teaching satisfaction. The integration of the MDT model with the flipped classroom teaching mode has shown remarkable effects in pediatrics and obstetrics and gynecology, both of which are conducive to cultivating students’ clinical thinking and practical abilities [15, 22]. It should be noted that there was no statistically significant difference in the theoretical knowledge assessment scores between the two groups of interns, which diverges from previous findings. This result may be attributed to the experimental group’s heavy learning workload, which involved extensive information searching and preparation of PowerPoint presentations prior to MDT discussions. Following the discussions, they summarized and organized relevant content using mind maps. In contrast, the control group had ample time to review theoretical knowledge after completing their clinical tasks, and some interns were preparing for postgraduate entrance examinations, thus placing greater emphasis on theoretical knowledge.

In this study, it was found through a questionnaire survey that the real MDT teaching model is significantly more effective than the traditional teaching model in multiple dimensions of teaching effectiveness. This finding aligns with a broader trend in educational research, where innovative, collaborative, and integrated teaching methods are increasingly recognized for their potential to improve learning outcomes [23]. The collaborative nature of the real MDT model may foster a more engaging and interactive learning environment [24]. Compared with the control group (55.17%), a substantially higher proportion (89.66%) of the experimental group reported that the real MDT teaching mode could stimulate learning interest. This is likely because the MDT discussion integrates multiple disciplines to provide a more comprehensive and contextualized learning experience, which is crucial for consolidating theoretical knowledge and expanding clinical knowledge. The experimental group had a significantly higher proportion of “yes” answers to these items, supporting this assertion. Moreover, the real MDT teaching model appears to be particularly effective in enhancing self-learning ability and team collaboration awareness, which are essential skills in today’s collaborative and interdisciplinary work environment. Most importantly, the significant improvement in clinical diagnosis and treatment reasoning ability (100% “yes” in the experimental group) underscores the potential of the real MDT teaching model to prepare students for real-world clinical scenarios [25]. Interns are fully involved in the process of patient diagnosis and treatment plan formation, which helps to cultivate imaging diagnostic thinking, provide valuable imaging content for clinical practice, and emphasize the ability to consider problems from a clinical perspective. These findings are consistent with studies advocating for experiential learning models in medical education, particularly in specialties that require the rapid integration of multimodal data.

The satisfaction of participants in the experimental group with the teaching mode has shown a statistically significant improvement. Unlike traditional methods where interns merely observe or engage in theoretical learning, this model allows them to actively participate in the diagnosis and treatment process of patients. This active involvement not only deepens their understanding of clinical practice but also cultivates a greater sense of achievement and professional identity. Within the real MDT environment, interns are not merely passive recipients of knowledge but also active contributors. Their insights are acknowledged, and they receive feedback from senior experts across various fields, which enhances their confidence and sense of belonging [24]. Moreover, discussing cases with professionals from different disciplines broadens the interns’ horizons. In summary, the high satisfaction of the experimental group can be attributed to the immersive, interactive, and affirming nature of the real MDT teaching model.

There are several limitations worth considering in this study. Firstly, the single-center design and moderate sample size may limit the generalizability of the teaching model. Secondly, although a 6-month intervention period is sufficient to observe skill acquisition, it may not capture long-term retention or behavioral changes in clinical practice. Thirdly, our hospital utilizes existing weekly MDT meetings for teaching purposes, requiring only the time commitment from trainees without additional financial investment. This provides favorable conditions for implementation. Medical institutions lacking established MDT meetings would require greater resource allocation to adopt this model. We are currently optimizing a more universally applicable MDT teaching program to enhance its scalability across diverse clinical settings. In addition, although self-reported survey data is informative, it is susceptible to reaction bias. Future research should include longitudinal follow-up, multicenter cohorts, and more objective indicators.

In summary, this study provides strong evidence that the real MDT teaching model helps improve the professional knowledge of radiology interns, achieve better teaching outcomes, and enhance overall satisfaction with teaching. The MDT-based teaching model addresses the limitations of traditional teaching by promoting interdisciplinary collaboration and clinical integration. In the future, it will be necessary to apply the real MDT teaching model to medical imaging interns to cultivate clinically oriented imaging talents.

## Supplementary information

ELECTRONIC SUPPLEMENTARY MATERIAL

## Data Availability

The data for this study cannot be shared. The original data can be uploaded to the editorial department for review.

## Abbreviations

CI: Confidence interval
MDT: Multidisciplinary team
