# Supplementary material for: Assessment of the real MDT teaching model in medical imaging internship: an educational-oriented study
Source: Insights Imaging. 2026 Aug 14;17:201. doi: 10.1186/s13244-026-02364-8 (PMC13476203; doi:10.1186/s13244-026-02364-8)
Supplement: Supplementary file 1 [file 13244_2026_2364_MOESM1_ESM.pdf]

Assessment of the Real MDT Teaching Model in Medical Imaging Internship: An Educational - oriented Study

ELECTRONIC SUPPLEMENTARY MATERIAL

Basic Information:

Male, 53 years old. Medical history: Urinary frequency, urgency for over 2 months, painless gross hematuria for 5 days throughout the entire process. Physical examination: Digital examination shows enlargement and hardness of the prostate gland. Laboratory test: PSA 34.13ng/ml.

Medical imaging images:

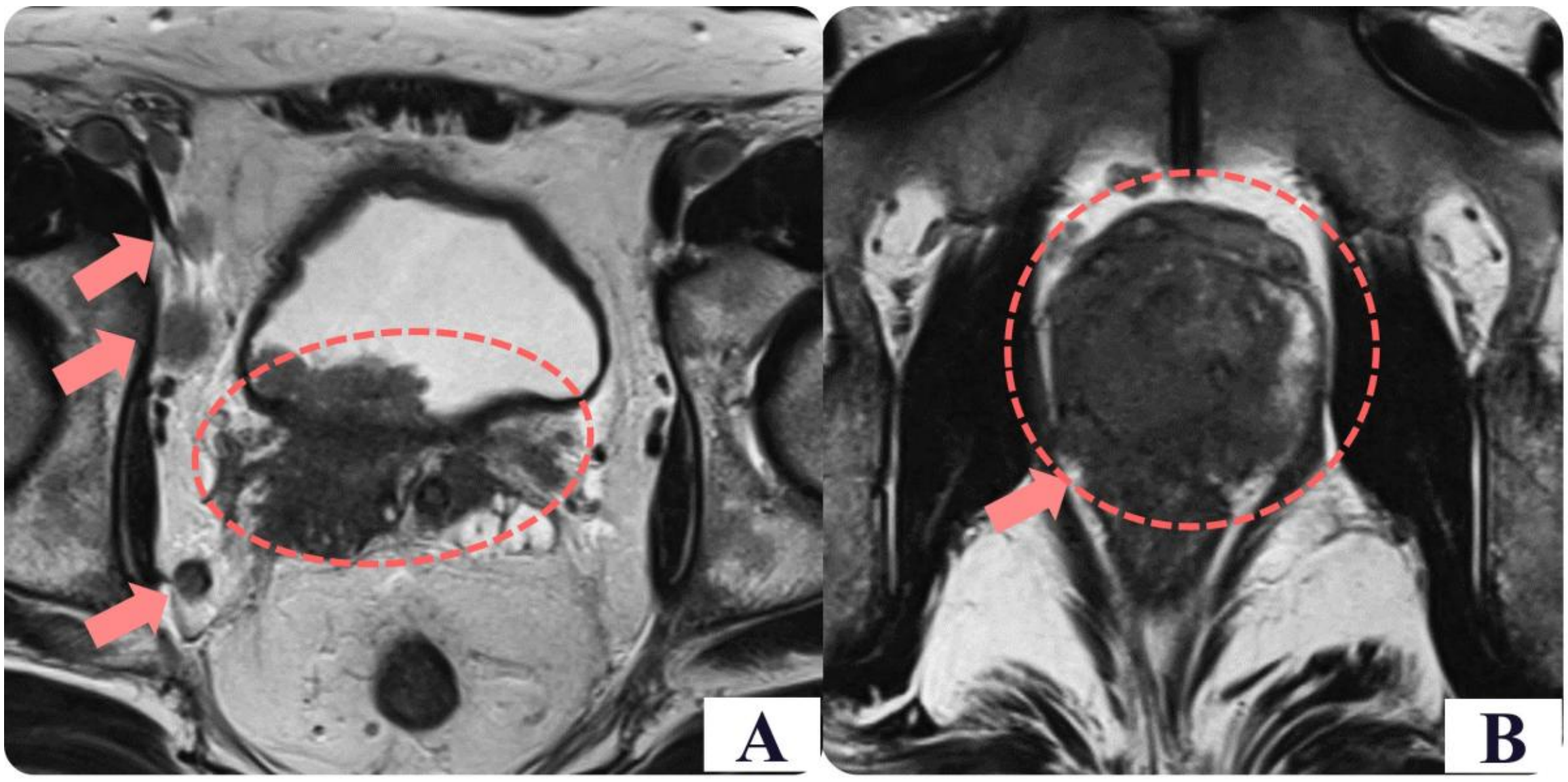

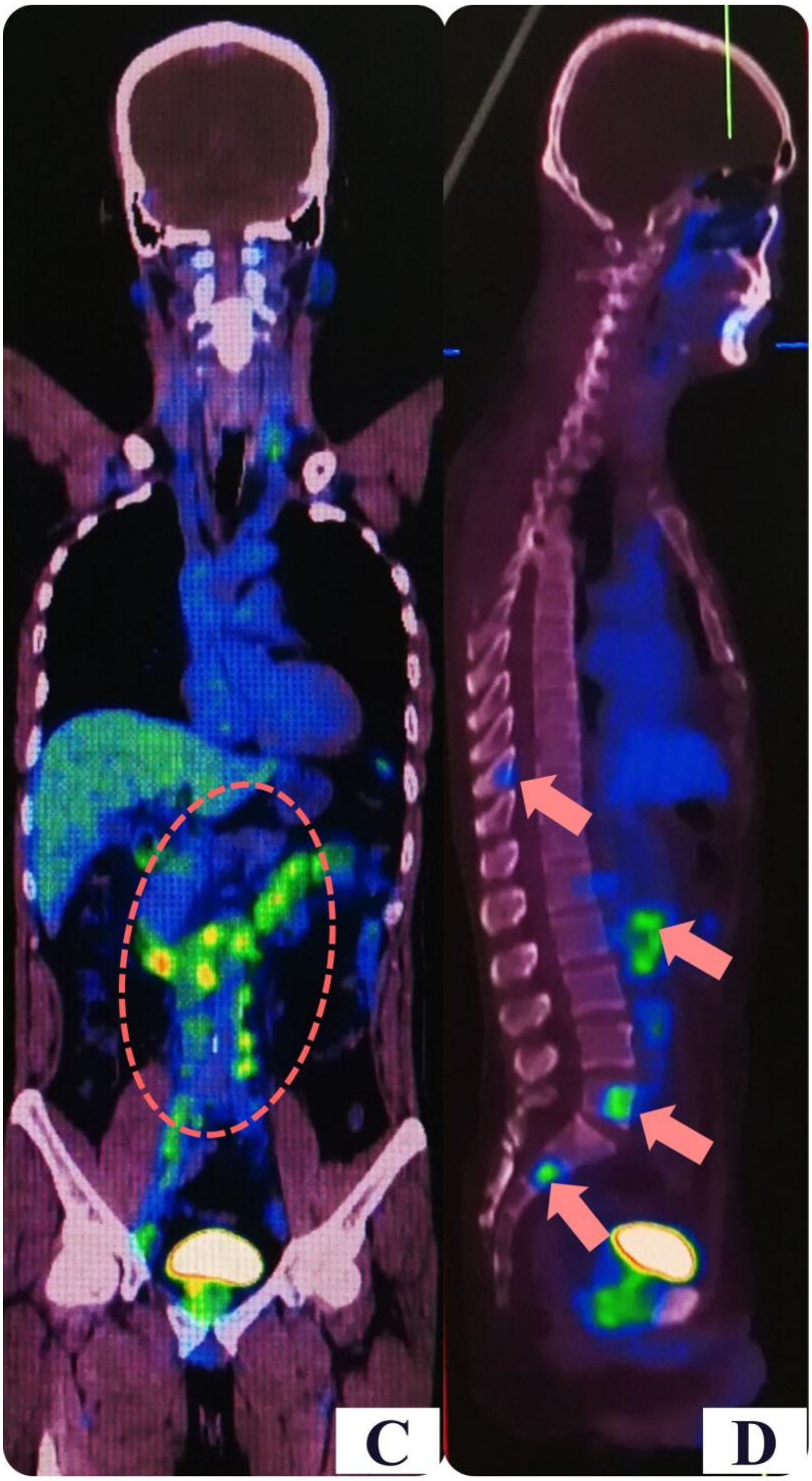

Supplementary Figure S1. Prostate cancer cases undergoing MDT cases. **A, B:** Axial T2WI show irregular clustered short T2 signal shadows in the central zone, transition zone, and bilateral peripheral zones of the prostate, with unclear boundaries, breaking through the capsule, involving the bilateral NVB, seminal vesicles, posterior bladder wall, anterior rectal fat space, multiple enlarged lymph nodes in the pelvic cavity and right iliac vessels (See red arrow and circle). **C, D:** Coronary and sagittal PSMA

PET/CT show increased uptake of multiple enlarged lymph nodes and bone lesions (See red arrow and circle).

MDT Conclusion: According to the radiological TNM classification (T4N1M1b, Stage IVB), the multidisciplinary urology team recommended endocrine therapy as the primary treatment strategy.

(https://v.wjx.cn/vm/ObCNrkZ.aspx)

Supplementary Figure S2. Access to the supplemental questionnaire. The QR code and the accompanying web link (provided below the code) grant access to the full text of the questionnaire employed for the subjective assessment.
